# Examining Sex Differences in Autism Heritability

**DOI:** 10.1001/jamapsychiatry.2024.0525

**Published:** 2024-04-17

**Authors:** Sven Sandin, Benjamin H. K. Yip, Weiyao Yin, Lauren A. Weiss, Joseph D. Dougherty, Stuart Fass, John N. Constantino, Zhu Hailin, Tychele N. Turner, Natasha Marrus, David H. Gutmann, Stephan J. Sanders, Benjamin Christoffersson

**Affiliations:** 1 Department of Medical Epidemiology and Biostatistics, Karolinska Institutet, Stockholm, Sweden; 2 Department of Psychiatry, Icahn School of Medicine at Mount Sinai, New York, New York; 3 Seaver Autism Center for Research and Treatment at Mount Sinai, New York, New York; 4 Jockey Club School of Public Health and Primary Care, The Chinese University of Hong Kong, Hong Kong SAR; 5 Institute for Human Genetics, University of California San Francisco; 6 Department of Psychiatry and Behavioral Sciences, University of California San Francisco; 7 Weill Institute for Neurosciences, University of California San Francisco; 8 Department of Genetics, Washington University School of Medicine, St Louis, Missouri; 9 Department of Psychiatry, Washington University School of Medicine, St Louis, Missouri; 10 Intellectual and Developmental Disabilities Research Center, Washington University School of Medicine, St Louis, Missouri; 11 Pediatric Institute, Emory University School of Medicine, Atlanta, Georgia; 12 Children’s Healthcare of Atlanta, Atlanta, Georgia; 13 Department of Neurology, Washington University School of Medicine, St Louis, Missouri; 14 Neurofibromatosis Center, Washington University School of Medicine, St Louis, Missouri; 15 Institute of Developmental and Regenerative Medicine, Department of Paediatrics, University of Oxford, Oxford, United Kingdom; 16 Department of Psychiatry and Behavioral Sciences, UCSF Weill Institute for Neurosciences, University of California, San Francisco; 17 Churney ApS, Copenhagen, Denmark

## Abstract

**Importance:**

Autism spectrum disorder (ASD) is a neurodevelopmental disorder more prevalent in males than in females. The cause of ASD is largely genetic, but the association of genetics with the skewed sex ratio is not yet understood. To our knowledge, no large population-based study has provided estimates of heritability by sex.

**Objective:**

To estimate the sex-specific heritability of ASD.

**Design, Setting, and Participants:**

This was a population-based, retrospective analysis using national health registers of nontwin siblings and cousins from Sweden born between January 1, 1985, and December 31, 1998, with follow-up to 19 years of age. Data analysis occurred from August 2022 to November 2023.

**Main Outcomes and Measures:**

Models were fitted to estimate the relative variance in risk for ASD occurrence owing to sex-specific additive genetics, shared environmental effects, and a common residual term. The residual term conceptually captured other factors that promote individual behavioral variation (eg, maternal effects, de novo variants, rare genetic variants not additively inherited, or gene-environment interactions). Estimates were adjusted for differences in prevalence due to birth year and maternal and paternal age by sex.

**Results:**

The sample included 1 047 649 individuals in 456 832 families (538 283 males [51.38%]; 509 366 females [48.62%]). Within the entire sample, 12 226 (1.17%) received a diagnosis of ASD, comprising 8128 (1.51%) males and 4098 (0.80%) females. ASD heritability was estimated at 87.0% (95% CI, 81.4%-92.6%) for males and 75.7% (95% CI, 68.4%-83.1%) for females with a difference in heritability estimated at 11.3% (95% CI, 1.0%-21.6%). There was no support for shared environmental contributions.

**Conclusions and Relevance:**

These findings suggest that the degree of phenotypic variation attributable to genetic differences (heritability) differs between males and females, indicating that some of the underlying causes of the condition may differ between the 2 sexes. The skewed sex ratio in ASD may be partly explained by differences in genetic variance between the sexes.

## Key Points

**Question:**

What are the sex-specific etiological origins of autism spectrum disorder?

**Findings:**

In this cohort study including 1 047 649 Swedish children, 12 226 (1.17%) received a diagnosis of autism spectrum disorder; heritability was estimated at 87.0% for males and 75.7% for females, a statistically significant difference.

**Meaning:**

These findings suggest that variation in the occurrence of autism spectrum disorder in the population differs between males and females, indicating that some of the underlying causes and prevalence of the condition may differ between the 2 sexes.

## Teaser

This cohort study analyzes sex-specific differences in heritability of autism spectrum disorder among individuals in Sweden.

## Introduction

Autism spectrum disorder (ASD) is a neurodevelopmental disorder present in 2% to 3% of children in the US.^1^ While the cause remains largely unknown, both common and rare genetic variants are associated with ASD.^2,3^ However, other factors are associated with trait variation, including sex.^4,5^ In order to understand the biological factors underlying ASD, it is important to elucidate the mechanisms underlying sex differences in ASD.^2^ It has been proposed that differences in prevalence between sexes is due to greater genetic variability in males (GVM).^6^

While a prior study using single-nucleotide variant (SNV) arrays^7^ showed similar SNV-based heritability in females and males,^8^ it should be noted that SNVs only capture a portion of the genetic variance.^9^ Thus, in contrast with the SNV-based study,^7^ a twin study^10^ using autistic traits as the outcome revealed higher heritability in males relative to females. However, as a result of the small sample size, the confidence intervals were wide and overlapping. More accurate estimates require population-based familial heritability analysis, which incorporates gene-gene interactions and familial aggregation.^11^ To address the heritability of ASD by sex, we leveraged a large population-based Swedish database to elucidate potential mechanisms underlying sex differences in ASD, calculating the narrow sense (ie, additive genetic) heritability using a sibling- and cousin-based study design.

## Methods

This cohort study was approved by the national Swedish ethics review board and followed the Strengthening the Reporting of Observational Studies in Epidemiology (STROBE) reporting guideline. No individual-level informed consent was required because all data used were anonymized and the study was conducted according to the Helsinki declaration.^45^

### Population

The study included all live-born, singleton children from Swedish parents born between January 1, 1985, and December 31, 1998, identified from the Swedish Medical Birth Register. The register links the children with their mothers and has covered 99% of all births nationwide since 1973.^12,13^ Father identification was obtained from the Swedish Multi-Generation Register,^14^ which includes all Swedish citizens aged 15 years or younger and their parents since 1947 (covering all individuals alive since 1961, when the register was computerized). See eAppendix 1 and eFigure 1 in Supplement 1 for more information on how individuals were included in the study.

### Analytical Data Set

To avoid biases due to differences in length of follow-up, and to optimize detection of ASD cases, we followed up all individuals up to age 19 years. Because twins are at increased risk of ASD^15^ and information about zygosity (necessary for heritability calculations in twins) was missing in our database, twins were excluded. To reduce genetic confounding and influences from missing values and parental identifiers, we included only children born to parents of Swedish origin. We further included only the first partner of the mother and father. We chose not to construct half-sibling families, which would rely on a questionable assumption about the correlation coefficient of the shared environmental effect. We restricted the analysis to the first 3 children of mothers in the parental and grandparental generations, further reducing the sample size, and specifically removed children where none of the grandparents were known.

Using a similar approach,^16^ we defined groups of dependent individuals as grandchildren of a set of grandparents yielding a pseudolikelihood where a child can be repeated at most twice. See eFigure 2, eFigure 3, eFigure 4, and eFigure 5 in Supplement 1 for examples of how offspring cousin pairs were identified.

### ASD Diagnoses

In Sweden, all children undergo regular medical and developmental examinations. At age 4 years, a mandatory developmental assessment (motor, language, cognitive, and social) is conducted. Children with suspected developmental disorders are referred to a specialized team in a child psychiatry unit or habilitation service. Diagnoses from specialist care are reported to the Swedish National Patient Register (NPR).^17^ The NPR includes all ASD inpatient diagnoses since 1987 and outpatient visits since 2001. The diagnoses are coded using the *International Classification of Diseases, Ninth Revision (ICD-9) *and *International Statistical Classification of Diseases and Related Health Problems, Tenth Revision (ICD-10)*. The NPR has been subjected to extensive validation efforts,^17,18^ including for autism diagnoses.^19^ Our database includes diagnoses in the NPR up to December 31, 2017, using *ICD-9* code 299A from 1987 and *ICD-10* code F84.0/1/3/5/8/9 from 1997.

### Covariates

**Figure 1.**
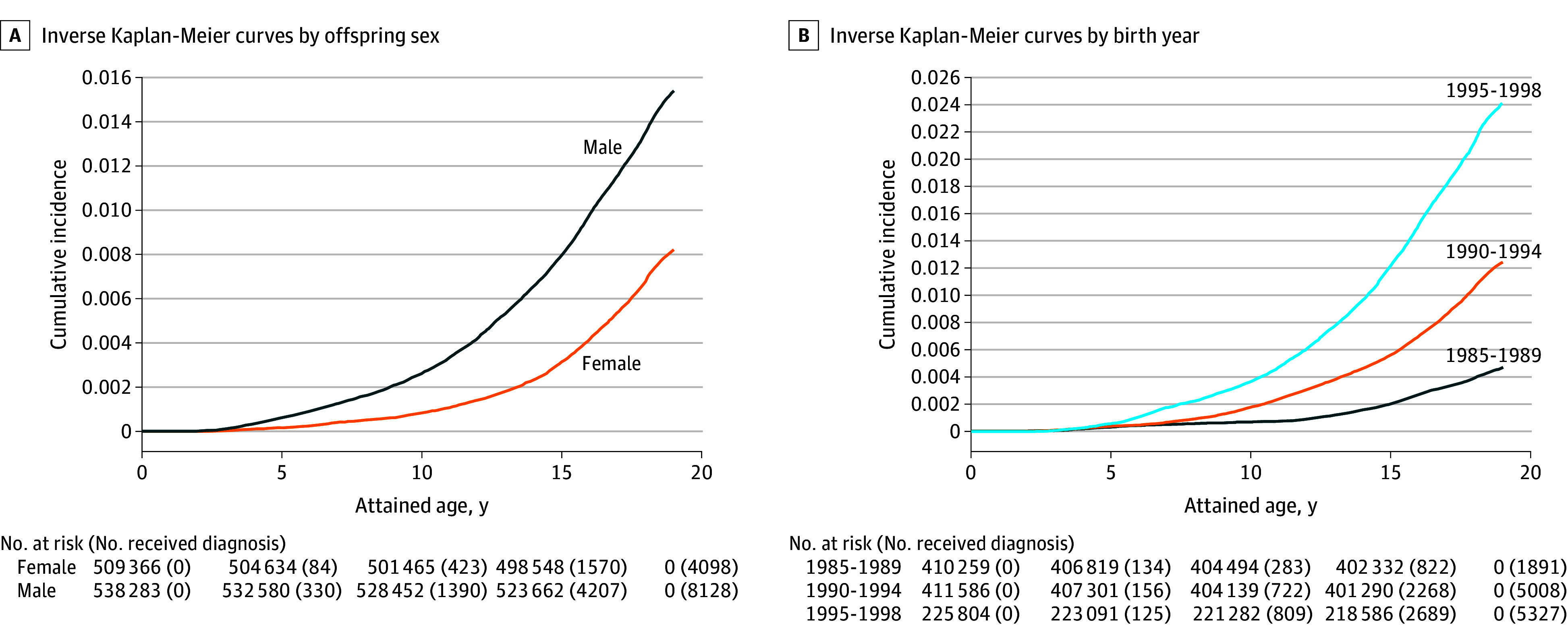
Inverse Kaplan-Meier Curves Depicting Cumulative Probability of Autism Spectrum Disorder, Overall and by Sex and Birth Year Kaplan-Meier curves based on incidence at 0, 5, 10, 15, and 19 years of age by sex (A) and by birth year (B).

Several variables may bias the results. Because the ASD incidence has increased rapidly in the last 15 to 20 years (Figure 1), we include birth year from the Medical Birth Register. Similarly, parental age is associated with increased risk of ASD and was considered as a covariate. Death and emigration information from the Sweden Total Population Register and Migration helped to adjust for censoring in the survival analyses. See eAppendix 2 in Supplement 1 for more information on simulating bias for heritability differences.

### Statistical Analysis

Kaplan-Meier depicts age-specific ASD incidence by 2 factors associated with ASD prevalence: sex and birth year. A 2-sided* P* <.05 was considered the threshold for statistical significance. Data analysis occurred from August 2022 to November 2023.

#### Sibling- and Cousin-Pair Correlations

Tetrachoric correlations were calculated to describe the overall pattern of genetic relatedness, an approach that relies on fewer but stronger assumptions regarding how the data were generated than required for the primary statistical models. A difference in correlation between male-male pairs and female-female pairs supports a sex difference in the genetic association with ASD risk. Tetrachoric correlation estimates the association of binary variables with underlying, normally distributed, continuous liabilities to develop a trait, which is not directly observable. Compared with the main analysis, this method does not utilize unpaired children or different-sex sibling pairs, which reduces the data by more than 50% and does not allow efficient adjustment for differences in prevalences (eg, between sexes and birth years).

#### Statistical Models

**Figure 2.**
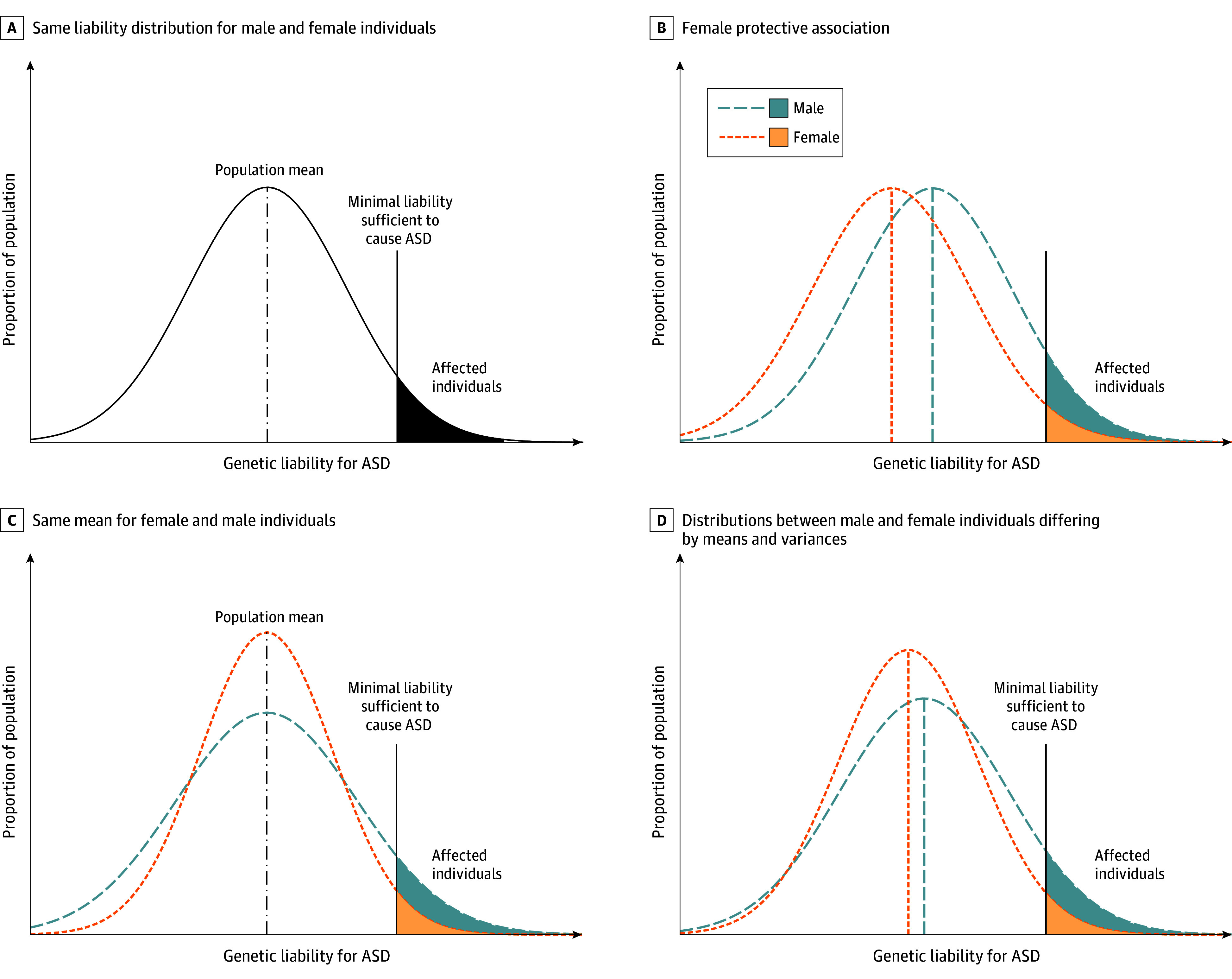
Possible Scenarios Under the Liability Threshold Model (LTM) The LTM proposes that, for dichotomous traits (eg, ASD), there is an underlying continuous distribution of liability for that trait in a population, typically assumed to be Gaussian. If an individual’s liability (x-axis) exceeds a specific diagnostic threshold (represented by the black vertical solid line), the individual will be considered affected. The figures assume 1 single diagnostic threshold. Panel A illustrates the conventional assumption that males and females share the same liability distribution, with the same population mean and variance. In panel B, a female protective effect is illustrated, where males and females have the same distribution for ASD liability, but the male distribution is shifted toward the singular diagnostic threshold, resulting in more males than females exceeding the diagnostic threshold. Panel C shows the same mean for both sexes, suggesting no female protective effect (but instead males have a greater variance) resulting in more males than females exceeding the diagnostic threshold. In panel D, the liability distributions between males and females differ by their means and variances. The female mean is shifted toward the diagnostic threshold, but proportionally more males cross the liability threshold than females due to a greater variance. ASD indicates autism spectrum disorder.

We modeled the probability of ASD using random-effect binary regression with a probit link, with fixed parameters to capture the liability thresholds and variance terms capturing the correlations due to inherited additive variance (Figure 2A and Figure 2B). The ASD status of an individual *j* in group *i* (ie, *Y_ij_*) was assumed to be positive when the liability (*η_ij_*) passes 0. The liability was considered a function of both fixed and random effects (*Y_ij_* = 1 when η_ij_ ≥ 0 and *Y_ij_* = 0 when η_ij_ < 0), where η_ij_ = x_ij_′β + σ_A_A_ij_ + σ_C_C_ij_ + ε_ij_. The fixed parameters β captured differences in ASD prevalence. The *x_ij_* values were assumed known fixed-effect design vectors, which, in our models, contained at most a fixed parameter for sex (male = 1 and female = 0), birth year (1985-1986, 1987-1989, 1990-1991, 1992-1994, and 1995-1998), and categories of paternal (≤28 years, 29-32 years, and ≥33 years) and maternal age (≤25 years, 26-30 years, and ≥31 years) at birth; and interactions between sex and birth year, paternal age, and maternal age. The categorical levels were based on quintiles for birth year and tertiles for parental age.

The random effects, A*_ij_* and *C_ij_*, were the additive genetic and shared environmental effects, respectively. A variance component, σ*_A_*, captured the correlation due to additive genetic variants inherited directly from the parents. The A*_ij_* had a correlation of 0.5 for 2 full siblings, 0.125 for 2 cousins, and 0 otherwise. The component, σ*_C_*, captured correlation from a shared childhood environment. The *C_ij_* had correlation of 1 for full siblings and 0 otherwise. Finally, the model included a residual random component (ε*_ij_*), capturing all other effects, uncorrelated for all individuals. We termed this as *residual*, instead of the commonly used *nonshared environment*, because the residual conceptually encompasses nonshared genetic factors as well.^20^ The random effects were assumed to follow a standard normal distribution. The scale parameters, σ*_A_* and σ*_C_*, were the primary focus in our analysis and gave the proportion of variance explained by each effect.

#### Sex-Specific Heritability Estimation

We were particularly interested in estimating the heritability by sex using the following equation:

σ*_A_*^2^ / (1 + σ*_A_*^2^ + σ*_C_*^2^).

To achieve this outcome, we allowed the components σ*_A_* and σ*_C_* to differ for males and females (Figure 2C and D). We computed the 2-sided 95% CIs^21^ using recently developed estimation techniques that, unlike previously used methods including the tetrachoric correlations, allowed us to control for more variables and to use individual data without splitting the data by sex, resulting in greater statistical power and a substantially reduced computational time.^22^

First, we fitted a model (M) to allow for a comparison with earlier heritability studies in the same population considering sex as the only fixed parameter (M1). Next, M3 additionally adjusted for birth year and interaction between sex and birth year. M5 contained the variables in M3 along with paternal and maternal age at birth and sex interactions. Paternal age is associated with an increased rate of de novo ASD-associated variants.^23,24^ Because de novo variants would not be included in the inherited additive genetic effects, we adjusted for these. M2, M4, and M6 were similar to M1, M3, and M5, but with sex-specific scale parameters. The supplementary models used an alternative parametrization for parental age (M7) and adjusted for gestational age (M9) (eAppendix 3 in Supplement 1). M8 and M10 were their counterparts with sex-specific scale parameters.

The new and faster technique to approximate the likelihood, an extension of an earlier method,^22^ allowed us to estimate the extended model and include larger families and more control variables than in previous studies using the same computational time (see online appendix). Analysis data sets were created using SAS software version 9.41 (SAS Institute). The statistical liability models were calculated using the R statistical software version 4.0.5 on a Linux server with the package pedmod version 0.2.4 (R Project for Statistical Computing).^25^ See eAppendix 4 in Supplement 1 for information on the computer code used.

#### Exploratory Analyses: Assessing Alternative Scenarios

We assumed the female-specific residual (capturing a multitude of factors) was underlying the difference in heritability rather than the greater genetic variance in males. We also assumed there was a single binary factor that explains the difference in heritability (eAppendix 5 in Supplement 1).

## Results

The analytical data set included a total of 1 047 649 individuals (eFigure 8 in Supplement 1) in 456 832 families (538 283 males [51.38%]; 509 366 females [48.62%]). By age 19 years, 12 226 individuals (1.17%) received an ASD diagnosis, including 8128 males (1.51%) and 4098 females (0.80%). The distribution of parental age and birth year was similar for males and females (Table 1). Sex- and birth year–specific prevalence are shown in the Kaplan-Meier plots, with higher cumulative ASD rates of diagnosis for males compared with females at age 19 years, as well as for later birth cohorts (1891 individuals [0.46%] for 19-year-olds born between 1985 and 1989 and 5327 individuals [2.36%] for 19-year-olds born between 1995 and 1998) (Figure 1).

**Table 1.** Characteristics of the Study Cohort

Characteristics and variables	Participants, No. (%) (N =1 047 649)	
	Males (n = 538 283)	Females (n = 509 366)
ASD diagnoses	8128 (1.51)	4098 (0.80)
ASD subcategories (% of ASD diagnoses)		
Autistic disorder	2338 (28.76)	963 (23.50)
Asperger	3743 (46.05)	1943 (47.41)
Other	2047 (25.18)	1192 (29.09)
ASD with intellectual disabilities (% of ASD diagnoses)	1664 (20.47)	759 (18.52)
Follow-up, median (IQR), y	19.0 (19.0-19.0)	19.0 (19.0-19.0)
Birth year		
1985-1989	211 227 (39.24)	199 032 (39.07)
1990-1994	211 185 (39.23)	200 401 (39.34)
1995-1998	115 871 (21.53)	109 933 (21.58)
Maternal age at delivery, y		
<20	8418 (1.56)	7851 (1.54)
20-29	301 561 (56.02)	284 987 (55.95)
30-39	216 028 (40.13)	205 165 (40.28)
40-49	12 272 (2.28)	11 357 (2.23)
≥50	4 (<0.00)	6 (<0.00)
Paternal age at delivery, y		
<20	2129 (0.40)	1989 (0.39)
20-29	213 016 (39.57)	201 420 (39.54)
30-39	275 982 (51.27)	261 764 (51.39)
40-49	44 358 (8.24)	41 589 (8.16)
≥50	2798 (0.52)	2604 (0.51)

Abbreviations: ASD, autism spectrum disorder.

### Sibling- and Cousin-Pair Correlations

Within–sibling-pair correlation was estimated at 0.45 (95% CI, 0.40-0.51) for brother pairs and 0.37 (95% CI, 0.29-0.47) for sister pairs. For cousin pairs, corresponding correlations were estimated at 0.10 (95% CI, 0.05-0.17) and 0.12 (0.03-0.23) but with overlapping male and female confidence intervals for both siblings and cousins. Thus, in the presence of genetic component only, and assuming 50.0% shared genetic load for siblings and 12.5% shared genetic load for cousins, we would expect 90.0% (ie, 2 × 45.0%) vs 75.0% heritability estimated from male and female siblings and 81.6% vs 98.4% heritability from male and female cousins (eTable and eFigure 6 in Supplement 1). Broadly, these summary statistics suggest a 15.0% higher genetic male variance using full siblings only, but a 16.8% lower genetic variance and 98.4% female heritability using cousins only. However, these correlations provide imprecise estimates of differences in heritability and do not account for differences in prevalence between sexes and birth year or adjust for other potential confounders.

### Heritability Estimates

Model parameters, variances, and goodness of fit are presented in Table 2 and a likelihood plot is exemplified in eFigure 7 in Supplement 1. For all models, the shared environmental effects were close to 0 and were not statistically significant. The overall heritability was estimated at 83.2% (95% CI, 79.3%-87.0%), after adjusting for birth year (M3). Adjusting for paternal and maternal age (ie, parameters potentially informative for increased variation due to de novo variants) resulted in a better model fit and a heritability estimate of 82.6% (95%CI, 78.7%-86.4%; M5). The conditional sex-specific heritability in M6 adjusting for birth year, sex and birth year interaction, and maternal and paternal age was estimated as 87.0% for males (95% CI, 81.4%-92.6%) and 75.7% for females (95% CI, 68.4%-83.1%), with a male vs female difference estimated at 11.3% (95% CI, 1.0%-21.6%). An alternative parametrization for parental age (M7 and M8) or adjustment for gestational age (M9 and M10) did not modify the estimates (Table 2).

**Table 2.** Variance Components, Heritability Estimates, and Goodness-of-Fit Model for the Probability of Autism Spectrum Disorder^a^

Model No.^b^	Random-effects model and model fit, fix model (No.)	−2× Likelihood	AIC (difference to above model)^c^	Heritability (*h^2^*) estimates (95% CI)			
				Overall	Male	Female	Difference male vs female
1	Sex	–158 639.20	158 647.20 (∆ = 0)	0.872 (0.696 to 0.910)	NA	NA	NA
2	As model 1	−158 633.53	158 645.54 (∆ = −1.66)	NA	0.894 (0.690 to 0.977)	0.786 (0.599 to 0.874)	0.108 (−0.082 to 0.262)
3	Sex + birth year + (sex × birth year)	−152 991.52	153 015.52 (∆ = −5630.02)	0.832 (0.793 to 0.869)	NA	NA	NA
4	As model 3	−152 987.10	153 015.10 (∆ = −0.42)	NA	0.875 (0.670 to 0.931)	0.765 (0.690 to 0.837)	0.110 (0.008 to 0.215)
5	Sex + birth year + paternal age + maternal age + (sex × birth year) + (sex × paternal age) + (sex × maternal age)	−152 779.58	152 819.58 (∆ = −195.52)	0.826 (0.787 to 0.864)	NA	NA	NA
6	As model 5	−152 775.06	152 819.06 (∆ = −0.52)	NA	0.870 (0.814 to 0.926)	0.757 (0.684 to 0.831)	0.113 (0.010 to 0.216)
7	As model 5 but alternative categorization of parental age	−152 901.87	152 941.87 (∆ = 122.81)	0.828 (0.789 to 0.865)	NA	NA	NA
8	As model 7	−152 897.47	152 941.47 (∆ = −0.40)	NA	0.871 (0.815 to 0.926)	0.760 (0.686 to 0.833)	0.111 (0.007 to 0.214)
9	Sex + birth year + paternal age + maternal age + gestational age + (sex × birth year) + (sex × paternal age) + (sex × maternal age) + (sex × gestational age)	−152 392.95	152 440.95 (NA)	0.825 (0.786 to 0.863)	NA	NA	NA
10	As model 9	−152 388.39	152 440.39 (NA)	NA	0.869 (0.813 to 0.924)	0.757 (0.682 to 0.829)	0.112 (0.010 to 0.216)

Abbreviations: AIC, Akaike information criterion; NA, not applicable.

^a^ All individuals were followed for autism spectrum disorder diagnosis up to age 19 years.

^b^ All models include 1 047 649 individuals (538 283 male and 509 366 female individuals) with a total of 12 226 cases of autism spectrum disorder (8128 male and 4098 female). All models additionally include variance terms reflecting any shared childhood environmental effect (sex specific in models 2, 4, 6, 8, and 10) being essentially 0 and not shown. The following variables were categorized into quintiles: birth year (1985-1987, 1987-1990, 1990-1992, 1992-1995, and 1995-1998), maternal and paternal age (≤25 years, 26-30 years, and >30 years), and gestational age (<37 weeks, 37-38 weeks, and >38 weeks). Models 1, 3, and 5 include shared variance terms and models 2, 4, and 6 include sex-specific variances for additive genetic effects and childhood shared environment. Models 7 and 8 had different maternal age groups (<32 years, 32-37 years, and >37 years) and paternal age groups (<35 years, 35-42 years, and >42 years).

^c^ The AIC and log likelihood cannot be compared between models 9 and 10 and models 1 and 8 because 1632 individuals with unknown gestational age were dropped. The difference in AIC is therefore deliberately omitted.

### Exploratory Analyses: Assessing Alternative Scenarios

Assuming that the female-specific residual is underlying the difference in heritability rather than greater genetic variance in males, the set of factors contributing to the female variance would have to be responsible for 37.0% of female ASD prevalence (eAppendix 5 in Supplement 1). If we instead assume there is a single binary factor that explains the difference in heritability, this factor must be very prevalent or very large. As a hypothetical example, if the sex difference in variance is due to a single unobserved factor among females, and it occurs in 6.0% of all females, it would have to increase the ASD risk by more than 10-fold. If it occurs in only 1.0% of females, it must increase the ASD risk by almost 40-fold (eFigure 8 in Supplement 1). Even with a prevalence as high as 6.0%, the factor would have to be sufficiently large to change the female ASD prevalence in a population from 0.5% to 5.5%.

## Discussion

To our knowledge, this cohort study is the first large family- and population-based study estimating the sex-specific associations of genetic and noninherited factors with ASD liability. Compared with previous studies,^16,26^ we used newly developed statistical estimation techniques, which allow for a detailed inclusion of fixed parameters and provide better adjustments for differences in prevalence by sex and birth year. Using a nationwide sample from Sweden with clinically ascertained diagnoses of ASD, we demonstrated a modest, but statistically significant, difference in heritability between sexes. This male-female difference in heritability, adjusting for differences in ASD prevalence by sex, birth year, and maternal and paternal age, was estimated at 11.3% (95% CI, 1.0%-21.6%). Additional adjustment for gestational age, a primarily environmental factor, did not modify the estimates. In agreement with previous studies,^16^ there was no support for shared environmental contributions, and the narrow-sense heritability in the overall population was estimated at approximately 80.0%, with the remaining 20.0% explained by individual-specific effects.

Our results indicate that a relatively larger portion of ASD diagnoses can be explained by additive genetic sources in males relative to females. It is also possible that females are less impacted by additive genetic sources, or are particularly vulnerable to other risk sources.

These other risk sources (referred to as residual risk in our models rather than the potentially misleading environmental term^20^) could theoretically arise from (1) classic environmental factors found in the built environment, (2) differences in the cultural environment leading to ascertainment or diagnosis differences, (3) genetic sources not inherited additively (eg, de novo variants), or (4) deficits in the model or its assumptions, such as interactions among genetic risk factors or genes and environment (eg, toxins,^27^ pollution,^28^ and maternal effects like type 1 diabetes or rheumatoid arthritis^29^). Additional clinical behavioral factors should also be considered, such as female-typical autism presentation (ie, female autism phenotype)^30^ or de novo variants and variation from rare variants not inherited additively or gene-environmental interactions. These effects could be examined in fully genotyped samples (where de novo variant carriers could be excluded before calculating sex-specific heritability) or using biological models to understand the potential gene-environment interactions. However, our sensitivity analyses suggested that the residual factors alone are not likely to explain ASD prevalence differences between the sexes. In addition, 1 factor associated with the rate of de novo variants is parental age, both paternal and maternal,^23^ which we adjusted for in our model, with only minor differences in heritability estimates.

Previous heritability estimates are based on statistical models where equal variances for males and females are assumed, resulting in equal heritability, and where sex is included as a fixed factor to adjust for differences in prevalence (Figure 1B). Such models agree with the theory of female protective effect (FPE),^31,32,33,34^ where females require a higher genetic threshold to be diagnosed with ASD, resulting in a lower prevalence compared with males. However, existing research shows mixed support for the FPE model.^11,34,35,36,37,38,39,40^ One implication of the FPE model is that familial ASD liability would be expected to aggregate asymptomatically in sisters of affected probands, who would incur elevated rates of ASD in their offspring. However, a previous population-based study largely overlapping with the current study did not support this possibility.^40^

Interestingly, a model allowing for sex-specific heritability offers an alternative explanation of the difference in ASD prevalence.^6,41^ If males are more vulnerable to additive genetic risk, this model would be consistent with the GVM, and other models proposing an interaction between sex and genetic risk.^34^ The 2 theories of FPE and GVM are, however, not mutually exclusive and could coexist (Figure 2D).

### Strengths and Limitations

This study has multiple distinguishing strengths. It includes data from a large, nationwide, population-based sample from a publicly financed and utilized health system ensuring study inclusion essentially free from selection biases. One may speculate that ASD data from Sweden, which is regarded as one of the most equal countries in the world^42^ may limit ascertainment biases due to sex. The extended 19-year follow-up minimized biases from differences in diagnosis age. Extending a previously published method^25^ to approximate the likelihood reduced the computing time, allowing the inclusion of larger families and more control variables to estimate variances by sex, without splitting the data set by sex.

Our study also has several limitations. Our results are based on individuals of Swedish origin only. In contrast with previous studies with greater restrictions,^40,43,44^ we only included the first 3 siblings or cousins born to each family. The approach of separating additive genetics, shared environment, and other contributions (ie, residual) relied on several untestable assumptions^11^ including that all individuals are ascertained independently. In addition, ASD diagnoses may be confounded by sex and temporal trends. This is possible given that the norms in diagnostic procedures and societal changes across time may have affected males and females differently, even though the sex ratio has remained stable.^34^ To address this, we included fixed parameters not only for sex and birth year, but also for their interactions. Additionally, there might be model misspecification. For example, we chose a probit link function over other possible link functions, whereas traditional liability threshold models rely on a Gaussian distribution. Also, both common and rare genetic variation are associated with ASD risk^3^; however, our study cannot determine whether common and rare variation equally contribute to sex differences in ASD risk. Additionally, we do not know to what extent the difference in estimated genetic variance can be attributed to phenotypic differences between males and females (eg, prevalence of ASD with and without comorbid conditions). Future studies could explore contributions of gene-environment interactions and underlying causes for estimated differences in heritability by incorporating ASD severity, cooccurring conditions, and age of first diagnosis to sex.

## Conclusions

Based on population-based data from Sweden, this cohort study found that genetic variability in ASD liability differs between males and females, indicating that some of the underlying causes of the condition may differ between the 2 sexes. The skewed sex ratio in ASD may, partly, be explained by differences in genetic variance between sexes. This discovery opens up new avenues for further research aimed at gaining a deeper understanding of the prevalence of ASD.

## Notes

Supplement 1. **eAppendix 1**. Illustration of How Individuals Were Included Into the Study **eFigure 1.** Sample 3-Generation Pedigree: Children Removed Due to Birth Order **eFigure 2.** Sample 2-Generation Pedigree: Children Removed Due to 1st-Partner-Restriction **eFigure 3.** Sample 3-Generation Pedigree: Children Duplicated in the Analysis Dataset **eFigure 4.** Plot of the Strength of the Unobserved Binary Exposure, ω, and the Prevalence of the Exposure (x-Axis ν), Which Gives the Observed Values Without the Binary Exposure **eFigure 5. **Flow Chart for Creating the Analytical Dataset **eAppendix 2.** Simulating Bias for the Heritability Difference **eAppendix 3.** Details of the Statistical Model **eAppendix 4.** Computer Code Used **eAppendix 5.** Results Under Different Modeling Assumptions **eTable.** Tetrachoric Correlations and 2-Sided 95% Confidence Intervals by Cousin and Sibling Male-Male and Female-Female Pairs **eFigure 6.** Genetic Correlations Between Family Members **eFigure 7.** Profile Likelihood for Differences in Heritability **eFigure 8.** Plot of ASD Prevalences for Females Who Are Exposed and Not Exposed (y-Axis) Plotted Against the Prevalence of the Exposure (x-Axis)

Supplement 2.
Data Sharing Statement
