# Supplementary material for: Examining Sex Differences in Autism Heritability
Source: JAMA Psychiatry. 2024 Apr 17;81(7):673–80. doi: 10.1001/jamapsychiatry.2024.0525 (PMC11024771; doi:10.1001/jamapsychiatry.2024.0525)
Supplement: Supplement 1. — eAppendix 1. Illustration of How Individuals Were Included Into the Study eFigure 1. Sample 3-Generation Pedigree: Children Removed Due to Birth Order eFigure 2. Sample 2-Generation Pedigree: Children Removed Due to 1st-Partner-Restriction eFigure 3. Sample 3-Generation Pedigree: Children Duplicated in the Analysis Dataset eFigure 4. Plot of the Strength of the Unobserved Binary Exposure, ω, and the Prevalence of the Exposure (x-Axis ν), Which Gives the Observed Values Without the Binary Exposure eFigure 5. Flow Chart for Creating the Analytical Dataset eAppendix 2. Simulating Bias for the Heritability Difference eAppendix 3. Details of the Statistical Model eAppendix 4. Computer Code Used eAppendix 5. Results Under Different Modeling Assumptions eTable. Tetrachoric Correlations and 2-Sided 95% Confidence Intervals by Cousin and Sibling Male-Male and Female-Female Pairs eFigure 6. Genetic Correlations Between Family Members eFigure 7. Profile Likelihood for Differences in Heritability eFigure 8. Plot of ASD Prevalences for Females Who Are Exposed and Not Exposed (y-Axis) Plotted Against the Prevalence of the Exposure (x-Axis) [file jamapsychiatry-e240525-s001.pdf]

Supplementary Online Content

Sandin S, Yip BHK, Yin W, et al. Examining sex differences in autism heritability.
JAMA Psychiatry. Published online April 17, 2024.
doi:10.1001/jamapsychiatry.2024.0525

eAppendix 1. Illustration of How Individuals Were Included Into the Study
eFigure 1. Sample 3-Generation Pedigree: Children Removed Due to Birth Order
eFigure 2. Sample 2-Generation Pedigree: Children Removed Due to 1st-Partner-Restriction
eFigure 3. Sample 3-Generation Pedigree: Children Duplicated in the Analysis Dataset
eFigure 4. Plot of the Strength of the Unobserved Binary Exposure, ω, and the Prevalence of the Exposure (x-Axis ν), Which Gives the Observed Values Without the Binary Exposure
eFigure 5. Flow Chart for Creating the Analytical Dataset
eAppendix 2. Simulating Bias for the Heritability Difference
eAppendix 3. Details of the Statistical Model
eAppendix 4. Computer Code Used
eAppendix 5. Results Under Different Modeling Assumptions
eTable. Tetrachoric Correlations and 2-Sided 95% Confidence Intervals by Cousin and Sibling Male-Male and Female-Female Pairs
eFigure 6. Genetic Correlations Between Family Members
eFigure 7. Profile Likelihood for Differences in Heritability
eFigure 8. Plot of ASD Prevalences for Females Who Are Exposed and Not Exposed (y-Axis) Plotted Against the Prevalence of the Exposure (x-Axis)

This supplemental material has been provided by the authors to give readers additional information about their work.

eAppendix 1. Illustration of how individuals were included into the study

The methods use to restrict the data set and form the families in this section are defined herin. Consider the following pedigree with two grandparents (G1 and G2), four children of the grandparents with their partners, which we denote as "parents" (P1 to P8), and nine grandchildren (C1-C9). A circle indicates a female and a square indicates a male.

eFigure 1. Sample 3-generation pedigree - Children removed due to birth order

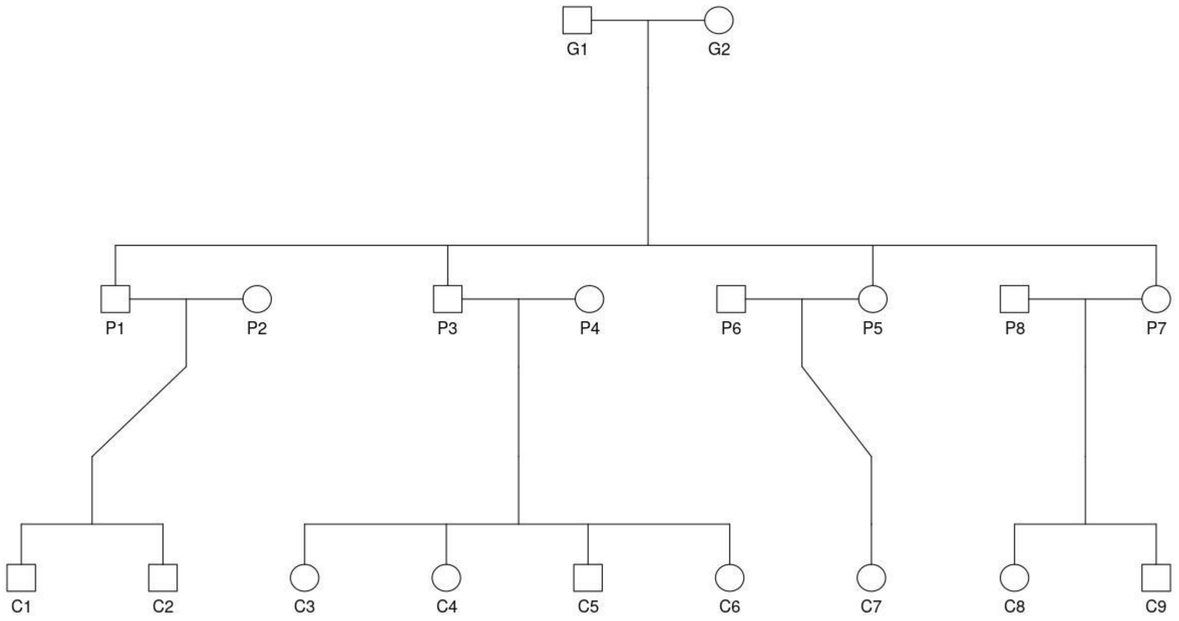

In this example, a lower number indicates a higher age. Thus, grandchildren C8 and C9 are removed as their mother is the fourth child. Moreover, grandchild C6 is removed because she is the fourth child of her mother. It should be noted that we count the birth order in the parent generation as the birth order of the mother. In the grandchild generation, we restrict the parents to be the first partner of each other. Thus, the birth order is the same regardless of whether one examines the mother or the father.

Next, we consider the following pedigree:

© 2024 Sandin S et al. *JAMA Psychiatry*

eFigure 2. Sample 2-generation pedigree - Children removed due to 1st-partner-restriction

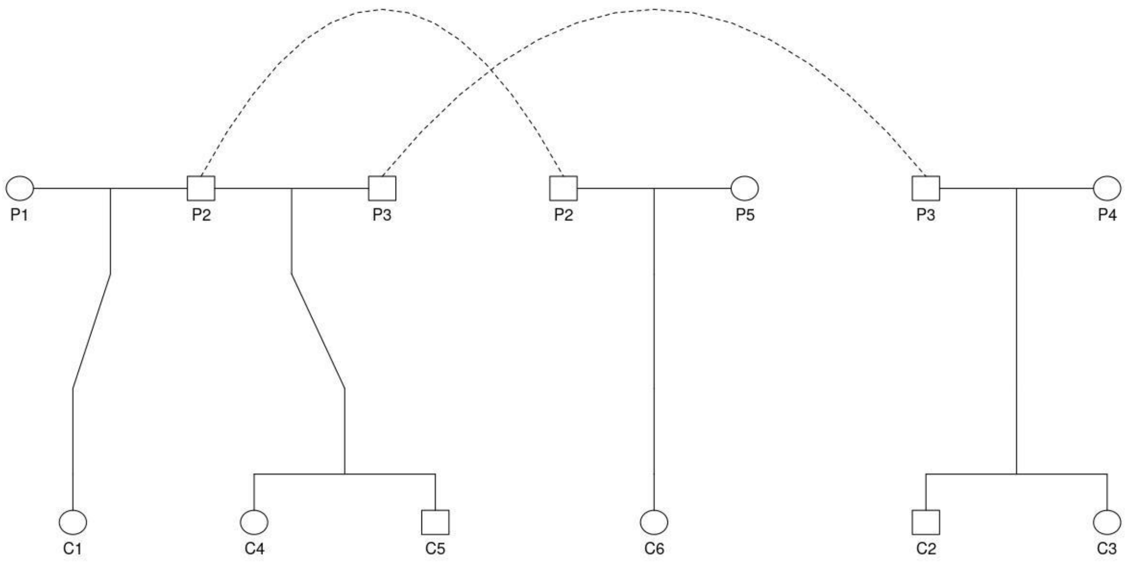

A dashed line indicates that it is the same individual. Here, we exclude grandchildren C4 and C5, as they are not children from the first partner of either of their parents. We also exclude C6 she is not a child of the first partner her farther, despite that she is the child of the first partner of her mother. Finally, we consider the way the grandchildren are possibly duplicated.

eFigure 3. Sample 3-generation pedigree - Children duplicated in the analysis dataset

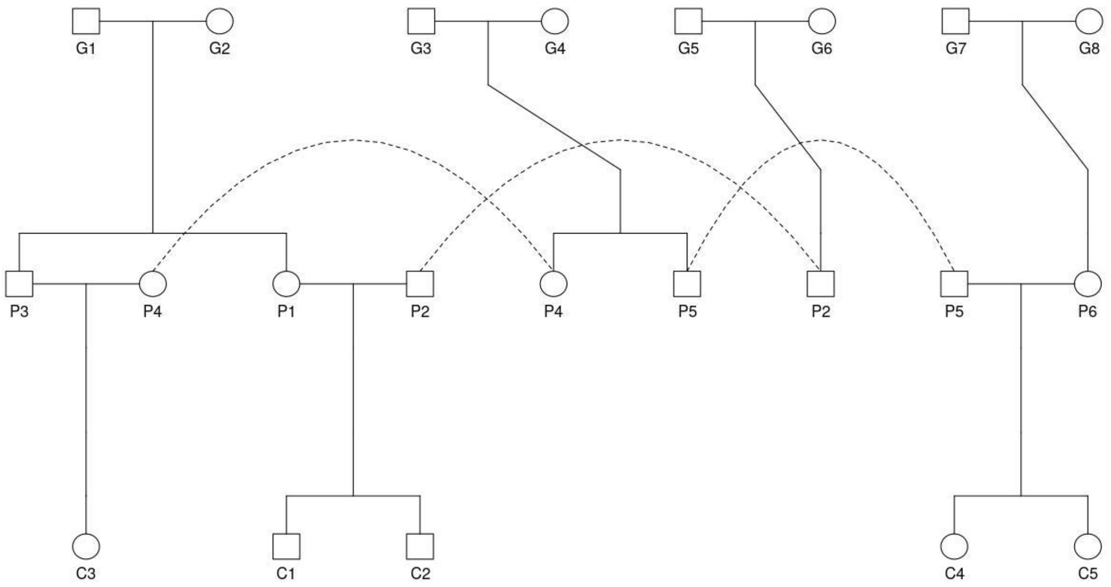

C1 and C2 are not duplicated, as they only have cousins on their mother's sides. C3 is duplicated, because she has cousins on both her mother's and father's side. C4 and C5 are not duplicated as they only have cousins on their father's side.

© 2024 Sandin S et al. *JAMA Psychiatry*

eFigure 4 Plot of the strength of the unobserved binary exposure, ω, and the prevalence of the exposure, x-axis ν, which gives the observed values without the binary exposure.

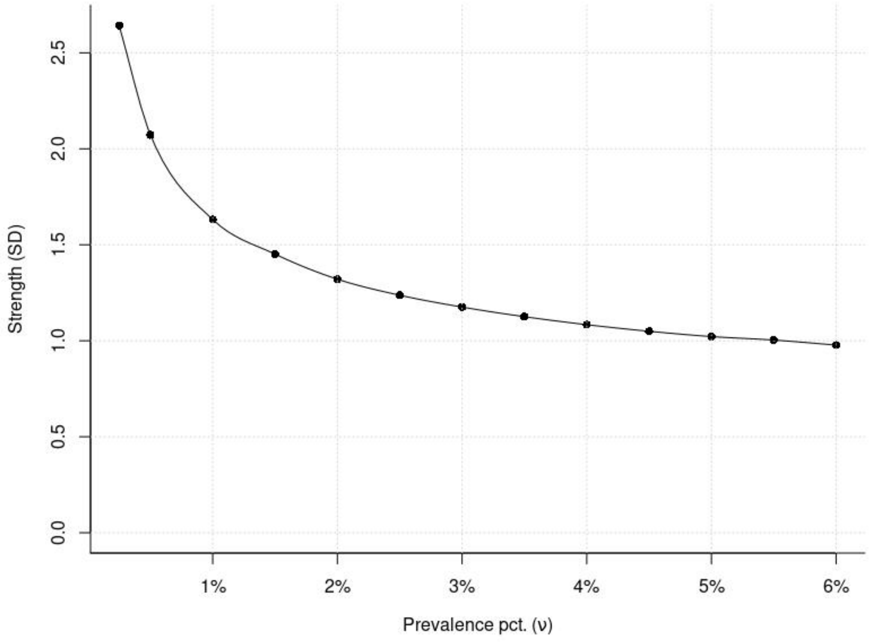

Note: The y-axis is divided by $\sqrt{(\sigma_{FA}^{2}+1)}$x so a unit is a standard deviation on the liability scale. Marks are computed values while the line is from a smoother.

© 2024 Sandin S et al. *JAMA Psychiatry*

eFigure 5 Flow chart for creating the analytical dataset

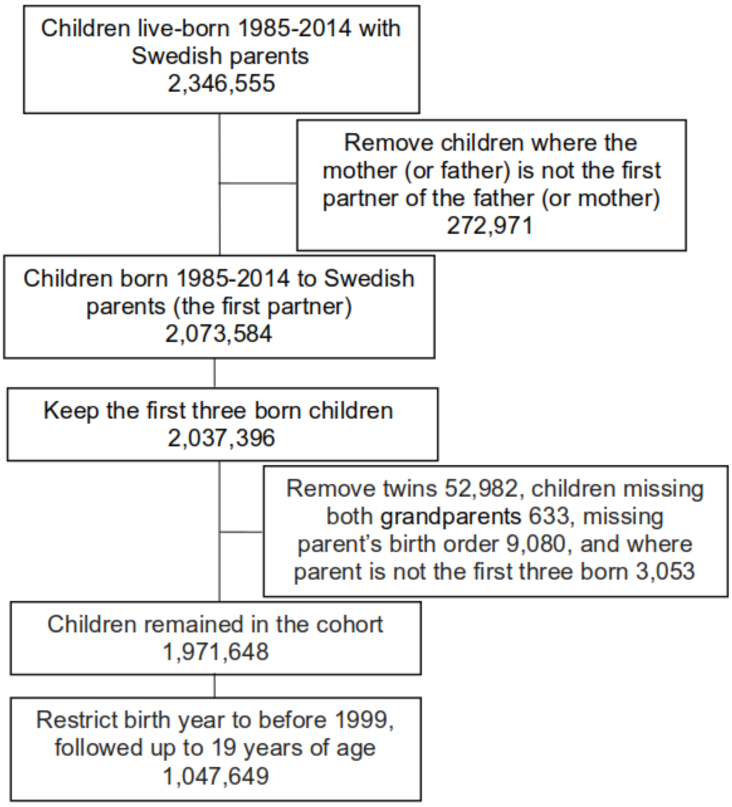

© 2024 Sandin S et al. *JAMA Psychiatry*

eAppendix 2. Simulating bias for the heritability difference

A possible concern is that the heritability difference could be biased because of the difference in prevalence between males and females. Bias may occur for two reasons in small samples. First, the maximum likelihood estimates of the variance parameters are biased for small sample sizes. In linear mixed models one can use restricted maximum likelihood estimates to avoid this but extensions to other generalized linear mixed models are not trivial. Second, heritability is a bounded parameter and could be biased in small samples for this reason. Importantly, the bias is negligible in either case for sufficiently large sample sizes.

To investigate whether this is a problem, we performed a simulation to check for possible bias. We simplified the data slightly to make it possible to do a large number of replications. We altered the numbers of families to see its effect on the heritability difference. Each family consists of two sets of siblings who are each other's cousins. Sex was assigned randomly for each child with a 51.4% chance of being a male. The male ASD prevalence is 1.51% and the female prevalence is 0.80% which matches Table 1. The heritability for males is 87.1% and 76% for females to match the model M6 results in Table 2.

We then sampled 100 data sets each consisting of 6,250 families (25,000 children), 25,000 families (100,000 children), and 100,000 families (400,000 children). The models were fitted and the heritability differences was computed. The models include a childhood environment effect, like in the analysis in the paper, even though the data generating distribution does not include one. The resulting bias estimates (average of the heritability differences less the true value) are -0.030, 0.011, and 0.004 with standard errors 0.028, 0.017, and 0.012 for 6,250, 25,000 and 100,000 families, respectively. None of this gives any evidence to suspect bias in our setting especially considering that we have more individuals than the 400,000 that are used here in the largest setting.

© 2024 Sandin S et al. *JAMA Psychiatry*

eAppendix 3. Details of the statistical model

Common liability threshold models with a childhood environment effect and an additive genetic effect assume that the ASD status of an individual j in group i is given by (**Eq. 0**)

$$Y_{ij} | A_{ij} = a, C_{ij} = c \sim \text{Bin}\left(p_{ij}(a, c)\right)$$

$$P\left(Y_{ij} = 1 \ \ | A_{ij} = a, C_{ij} = c\right) = p_{ij}(a, c) = \Phi\left(x_{ij}^{\top} \beta + \sigma_A a + \sigma_E c\right)$$

where A_{ij} and C_{ij} are standard normally distributed random effects for the additive genetic effect and the childhood environment effect, respectively, $\text{Bin}(p)$ denotes a binary variable with probability p of being one and Φ is the standard normal distribution's cumulative density function. The model can also be written as (**Eq. 1**),

$$Y_{ij} = \begin{cases} 1 & \eta_{ij} \geq 0 \\ 0 & \eta_{ij} < 0 \end{cases}$$

$$\eta_{ij} = x_{ij}^{\top} \beta + \sigma_A A_{ij} + \sigma_C C_{ij} + \epsilon_{ij}$$

where ϵ_{ij} is an independent standard normally distributed random variable for the model residual capturing contributions not covered by the other two terms and η_{ij} is the "liability". The additive genetic correlation coefficient, A_{ij}s, is 0.5 for full siblings and 0.125 for cousins. We assume that the correlation coefficient C_{ij}s is one for children with the same mother and father and zero otherwise, like cousins. The shared environment effect does not take into account gaps in ages between siblings nor their sexes, a limitation in genetic epidemiological studies when using family data beyond twins data. ϵ_{ij} is uncorrelated between all individuals. The x_{ij}s are known fixed effect design vectors, which in the models

© 2024 Sandin S et al. *JAMA Psychiatry*

contain at most a fixed parameter for sex (male=1, female=0), birth year ([1985,1986), 1987,1989), [1990,1991), [1992,1994), [1995,1998]) the categories of paternal (≤28, 29-32, ≥33) and maternal age (≤25, 26-30, ≥31) at birth, and interactions between sex and the other variables. The categorical levels were based on quintiles for birth year and tertiles for parental age. The scale parameters, σ_A and σ_C, are of main interest in this analysis, which provides the proportion of variance explained by each effect. We are particularly interested in estimating the heritability (**Eq. 2**):

$$\frac{\sigma_A^2}{1+\sigma_A^2+\sigma_C^2}$$

Note that the scale in the model is not identifiable, so we set the variance of one of three random effects as is customary. To see that the scale is not identifiable, we can define a $\varphi>0$ and then (**Eq. 3**)

$$Y_{ij}=\begin{cases}1 & \tilde{\eta}_{ij}\geq 0\\ 0 & \tilde{\eta}_{ij}<0\end{cases},\tilde{\eta}_{ij}=\varphi>0$$

is the same model. The above states that the latent score, the liability, has a residual term with variance φ^2, which was fixed to 1 beforehand. The implication of the equivalence is that we can only determine whether the additive genetic effect (or the shared environment) has a variance that is larger or smaller relative to the other two components but we cannot estimate the magnitude and we cannot estimate the total variance as φ is not identifiable.

We next extend the model to allow for different genetic variances through sex specific scale parameters. Using $s_{ij}\in\{M,F\}$ to represent the sex of individual j in group i, the model we estimate is (**Eq. 4**),

$$Y_{ij}=\begin{cases}1 & \eta_{ij}\geq 0\\ 0 & \eta_{ij}<0\end{cases}$$

© 2024 Sandin S et al. *JAMA Psychiatry*

$$\eta_{ij} = x_{ij}^{\top} \beta + \sigma_{s_{ij}A} A_{ij} + \sigma_{s_{ij}C} C_{ij} + \epsilon_{ij}$$

which now includes four rather than two scale parameters, σ_{MA}, σ_{MC}, σ_{FA} and σ_{FC}.

One challenge with this model is that the marginal log likelihood is intractable where each likelihood factor consists of an integral in which the number of variables is equal to the family size (the number of grandchildren). However, an efficient importance sampler can be applied to approximate the likelihood, and can also be extended to approximate the gradient as well[1,2].

The main interest is the difference in the proportion of variance attributable to the genetic effect between the two sexes. Particularly, we would like to test the null hypothesis, in which that the heritabilities are identical against the two-sided alternative that they are not(**Eq 5**)

$$H_0 : \frac{\sigma_{MA}^2}{1 + \sigma_{MA}^2 + \sigma_{MC}^2} = \frac{\sigma_{FA}^2}{1 + \sigma_{FA}^2 + \sigma_{FC}^2}$$

Some caution is needed for the fixed effect parameters. The marginal probability in the above model of an individual having ASD is (**Eq. 6**):

$$P(Y_{ij} = 1) = \Phi\left(\frac{x_{ij}^{\top} \beta}{\sqrt{1 + \sigma_{s_{ij}A}^2 + \sigma_{s_{ij}C}^2}}\right)$$

The implication is that, for instance, a higher scale parameter for the additive genetic effect yields a marginal probability closer to 50% unless the increase can be offset by fixed effects. However, this offsetting by fixed effects is not desired as we are only interested in the relative size of the genetic effect which creates dependence between individuals. To solve the fixed effect offsetting issue, we added interactions between sex and all other covariates in each model we estimated.

© 2024 Sandin S et al. *JAMA Psychiatry*

Using the above strategy, we created a sequence of models: First, we fitted a model (M1) to allow for a comparison to earlier heritability studies in the same population considering sex as the only fixed parameter. The next model (M3) adds to model M1 and additionally adjusts for birth year using a five-level dummy and sex-by-birth year interaction. M5 then contains the variables in M3 along with paternal and maternal age at birth as three-level dummies along with sex interactions. Paternal age is known to result in de novo mutations of ASD-associated mutations[3]. Since de novo mutations would not be included in the directly inherited additive genetic effects, we also wanted to adjust for these. Therefore, M2, M4 and M6 are similar to M1, M3 and M5, but with sex-specific scale parameters.

We fitted four additional supplementary models. M7 is like M5 but were the interior limits are the 75% and 95% quantile of maternal and paternal age conditional on the child having an ASD diagnosis. This yields the groups [14,35) [35,43) [43,∞) for paternal age and [14,32) [32,38) [38, ∞) for maternal age. These more extreme groups were used to potentially capture relations between paternal age and de novo mutations better among other things. M9 extends M5 with a dummy for whether the gestational week is less than 37, a dummy for whether the gestational week is 37 or 38 and their interaction with sex. M8 and M10 are the counterparts of M7 and M9, respectively, but with sex specific scale parameters.

Finally, we computed a profile likelihood-based confidence interval for the difference between the sex specific heritabilities (**Eq. 7**):

$$\frac{\sigma_{MA}^2}{1+\sigma_{MA}^2+\sigma_{MC}^2}-\frac{\sigma_{FA}^2}{1+\sigma_{FA}^2+\sigma_{FC}^2}$$

We constructed the (pseudo) likelihood by creating a cluster for each pair of grandparents as

described in section described above.

References

1. Pawitan Y, Reilly M, Nilsson E, Cnattingius S, Lichtenstein P. Estimation of genetic and environmental factors for binary traits using family data. Stat Med. 2004 Feb 15;23(3):449–465. PMID: 14748038
2. Christoffersen B. pedmod: Package for mixed probit models in R [Internet]. 2022 [cited 2022 Sep 13]. Available from: https://cran.r-project.org/package=pedmod
3. Iossifov I, O'Roak BJ, Sanders SJ, Ronemus M, Krumm N, Levy D, Stessman HA, Witherspoon KT, Vives L, Patterson KE, Smith JD, Paeper B, Nickerson DA, Dea J, Dong S, Gonzalez LE, Mandell JD, Mane SM, Murtha MT, Sullivan CA, Walker MF, Waqar Z, Wei L, Willsey AJ, Yamrom B, Lee Y ha, Grabowska E, Dalkic E, Wang Z, Marks S, Andrews P, Leotta A, Kendall J, Hakker I, Rosenbaum J, Ma B, Rodgers L, Troge J, Narzisi G, Yoon S, Schatz MC, Ye K, McCombie WR, Shendure J, Eichler EE, State MW, Wigler M. The contribution of de novo coding mutations to autism spectrum disorder. Nature. 2014 Nov 13;515(7526):216–221. PMCID: PMC4313871

eAppendix 4. Computer code used

We provide a simplified example of calling the code in the pedmod package to estimate the models described in this report. We strongly encourage the reader to review the vignette in the package which provides both formulas and more detailed explanations.

Estimating the models starts with a syntax like the following in R:

```
library(pedmod)
ll_terms <- pedigree_ll_terms_loadings(dat, max_threads = 4L)
```

This creates the C++ object which is used to evaluate the log likelihood and the gradient of it. The parameter "dat" needs to be a list of lists and "max_threads" indicates the greatest number of CPU threads to use in the computation. Each list in "dat" is a cluster (family in this case), assumed to be possibly marginally dependent. It contains element "y" with the binary outcomes, a design matrix "X" for the fixed effects (note that the user will have to add an intercept), a design matrix "Z" for the individual specific random effects (in this paper an intercept and a male dummy) and a list correlation matrices called "scale_mats". The pedmod package can have an arbitrary number and types of effects (hence correlation matrices). In this paper we have two correlation matrices per cluster: a correlation matrix for the additive genetic effect and a correlation matrix for the shared childhood environment.

Thus, the model can then be fitted by calling:

```
start <- pedmod_start_loadings(ll_terms, data = dat)
opt_res <- pedmod_opt(
    ll_terms, par = start$par, maxvls = 25000L, minvls = 5000L,
    abs_eps = 0, rel_eps = 1e-8, n_threads = 4L
)
```

The first call, "start", uses a heuristic approach to find the starting values while the second call does the final optimization. "maxvls" is the maximum number of samples to use per cluster in the importance sampler and minvls is the minimum number. "abs_eps" and "rel_tol" are respectively the absolute and relative convergence thresholds for the importance sampler and "n_threads" is the number of threads to use.

© 2024 Sandin S et al. *JAMA Psychiatry*

If the clusters have weights, then one can make a call like:

```
start <- pedmod_start_loadings(ll_terms, data = dat,
      cluster_weights = c_weights)
opt_res <- pedmod_opt(
    ll_terms, par = start$par, maxvls = 25000L, minvls = 5000L,
    abs_eps = 0, rel_eps = 1e-8, n_threads = 4L,
    cluster_weights = c_weights, vls_scales = sqrt(c_weights)
)
```

“cluster_weights” is used to pass on the weights and “vls_scales' ' sets the maximum and minimum number of samples proportional to the passed argument on a cluster level. Using the square root of the weights usually works well for clusters of roughly equal size.

It is important to check that the gradient at the result from “pedmod_opt()” is roughly a zero vector. The gradient can be approximated with the “eval_pedigree_grad()” function with similar arguments as “pedmod_opt()”.

Subsequent inference can be performed using profile likelihood. This can either be performed using “pedmod_profile()” to compute a profile likelihood based confidence interval for one of the parameters or “pedmod_profile_nleq()” for a function of the parameters (such as the heritability difference). Finally, the “use_tilting” argument may be worthwhile using in some applications. When true, minimax tilting is used which may greatly reduce the noise from the importance of sampler at the cost of longer computation times at a fixed number of samples.

© 2024 Sandin S et al. *JAMA Psychiatry*

eAppendix 5. Results under different modeling assumptions

What if the difference in heritability does not represent a true underlying etiology, but is instead caused by model miss-specification (e.g., due to unmeasured factors)? Below, we propose two scenarios corresponding to two alternative causes (C1 and C2). In a third proposal (C3), we examine the support for FPE within the current model. Because of sex interactions in model M3 to M6, we work with numbers deduced from the prevalence. Although these are essentially identical M1 and M2, the interactions are added: Otherwise, the birth year effect would become smaller for the males with greater variance (see Equation 6), which would create nonsensical artifacts.

C.1. Proposal 1: Estimated heritability difference is due to an increased female residual variance

In this section, we investigate how much the ASD prevalence (i.e., the marginal rate of the females would drop in a hypothetical scenario), where (1) the heritability difference between sexes is solely driven by an additional residual term for females and (2) if such additional effects are eliminated. The residual term may capture different influences (e.g., unshared environmental effects as well as de novo mutations). The shared environment effect C_{ij}, is always tiny in the models we fitted and was removed for reasons of simplicity. Thus, we consider a simplified model for the females where we have decomposed the residual into two terms. The liabilities for the females are then:

$$\eta_{ij}=\beta_F+\sigma_{FA}A_{ij}+\sqrt{1-\rho^2}\xi_{ij}+\rho\epsilon_{ij}$$

where A_{ij}, ξ_{ij} and ϵ_{ij} are standard normally distributed, ρ is a scalar, $\rho\in[0,1]$. ξ_{ij} is a hypothetical residual effect unique for females, A_{ij} is for the genetic effect, and ϵ_{ij} is the residual effect common for both males and females. Consequently, the male liability (with $\xi_{ij}=\beta_M+\sigma_{MA}A_{ij}+\rho\,\epsilon_{ij}$

The female prevalence in the simplified model and the heritability are, respectively,

$$p_F=\Phi\left(\frac{\beta_F}{\sqrt{1+\sigma_{FA}^2}}\right), h_F^2=\frac{\sigma_{FA}^2}{1+\sigma_{FA}^2}$$

If we remove the hypothetical female residual effect, $\xi_{ij}=0$, we have

$$p_F=\Phi\left(\frac{\beta_F}{\sqrt{\rho^2+\sigma_{FA}^2}}\right), h_F^2=\frac{\sigma_{FA}^2}{\rho+\sigma_{FA}^2}$$

As an example, we take the heritability to be 0.76 for the females which matches Table 2 with a prevalence of 0.80% which matches Table 1. We then find the initial parameters by solving:

$$\sigma_{FA}^2=\frac{h_F^2}{1-h_F^2}, \beta_F=\Phi^{-1}(p_F)\sqrt{1+\sigma_{FG}^2}$$

which yields $\sigma_{FA}=\sqrt{3.167}$ and $\beta_F=-4.917$. Finally, to match the males' heritability of 0.87

© 2024 Sandin S et al. *JAMA Psychiatry*

(which match Table 2) we solve:

$$\rho^2 = \left(h_F^{-1} - 1\right)\sigma_{FA}^2$$

using $h_F{}^2 = 0.87$. This gives $\rho^2 = 0.4731$.

To reason about the plausibility of such a female residual effect, we consider the case where it is completely eliminated. If the female residual effect could be entirely removed from the female population, it would result in a drop of the already low female prevalence with 37%, i.e. from 0.8% (see Table 1) to 0.5%.

C.2. Proposal 2: Estimated heritability difference is due to a single binary female factor

In this section, we assume that there is a single unmodelled binary exposure (e.g., rare variants or a unique environmental risk factor) and ask how strong this exposure would need to be to explain the difference in heritability between males and females, assuming a fixed prevalence of the exposure.

We start with an expression for the liabilities for females

$$\eta_{ij} = \beta_F + \sigma_{FA} A_{ij} + \epsilon_{ij}$$

with a heritability of $h_F{}^2 = 0.87$ (the estimate for males) and a prevalence of $p_F = 0.008$ (similar to that of females). This implies $\sigma_{FA} = \sqrt{6.6923}$ which we will use to fix σ_{FA} in the subsequent examples.

Assume that the underlying truth used to generating the observed data is:

$$\eta_{ij} = \widetilde{\beta}_F + \omega D_{ij} + \sigma_{FA} A_{ij} + \epsilon_{ij}, \omega \geq 0, D_{ij} \sim \text{Bin}(v), v \in (0,0.5)$$

but we do not observe the binary exposure D_{ij}. The question then is what combinations of the strength of the effect, ω, and the population prevalence of the unobserved binary exposure, v, can give the observed values $\hat{\sigma}_{FA} = \sqrt{3.167}$ ($h_F{}^2 = 0.76$) and $\hat{\beta}_F = -4.917$ ($p_F = 0.008$ when $h_F{}^2 = 0.76$) in the model where we do not include D_{ij} as a fixed effect (i.e. a misspecified model).

As an example, we include families consisting of only two siblings for simplicity and consider the limiting case with an infinitely large sample (so we can compute the expected fraction of cases in each combination of the binary exposure and the outcome). Then we find the (ω, v) values that yields $\hat{\sigma}_{FA} = \sqrt{3.167}$ and $\hat{\beta}_F = -4.917$ when D_{ij} is not included in the model and $\sigma_{FA} = \sqrt{6.6923}$.

The full procedure is:

- Fix $\sigma\, FA = \sqrt{6.6923}$.
- Find (ω, v) that yields $\hat{\sigma}\, FA = \sqrt{3.167}$ and $\hat{\beta} F = -4.917$ where for given (ω, v) where find the βF in the true model so the prevalence matches $p_F = 0.008$ in a true model for consistency.

This yields the eFigure 4 and illustrates the exposure level of a single binary factor, affecting women, and its effect on ASD prevalence, assuming other factors to be fixed, to level out the observed difference in ASD prevalence between sexes. We standardize the y-axis by dividing with √ σ2FA so the units on the y-axis are standard deviations (SD) on the liability scale. The plot shows that an exposure with a prevalence of 1%, or less, in the total population needs to move the liability by at least 1.63 SD if present. As an example, this represents a change in prevalence from 0.62% to 19.2%, i.e.

$\Phi(-2.5) = 0.0062$ to $\Phi(-0.87) = 0.192$, if an individual is exposed (eFigure 8).

eTable. Tetrachoric correlations and 2-sided 95% confidence intervals by cousin and sibling male-male and female-female pairs

M-M sibling	M-F sibling	F-F sibling	M-M cousin	M-F cousin	F-F cousin
0.447<br>(0.401, 0.508)	0.429<br>(0.382, 0.480)	0.373<br>(0.293, 0.472)	0.102<br>(0.047, 0.171)	0.107<br>(0.053, 0.162)	0.123<br>(0.035, 0.234)

M-M: Male-Male; F-F: Female-Female; M-F: Male-Female.
Note: The confidence intervals are based on non-parametric bootstraps where we sampled individuals. The correlations were calculated using R-package mvtnorm to evaluate the CDF of bi-variate normal distribution and the optimization was done using the optim function from the stats package. The bootstrap packages was used for the confidence intervals.

eFigure 6. Genetic correlations between family members

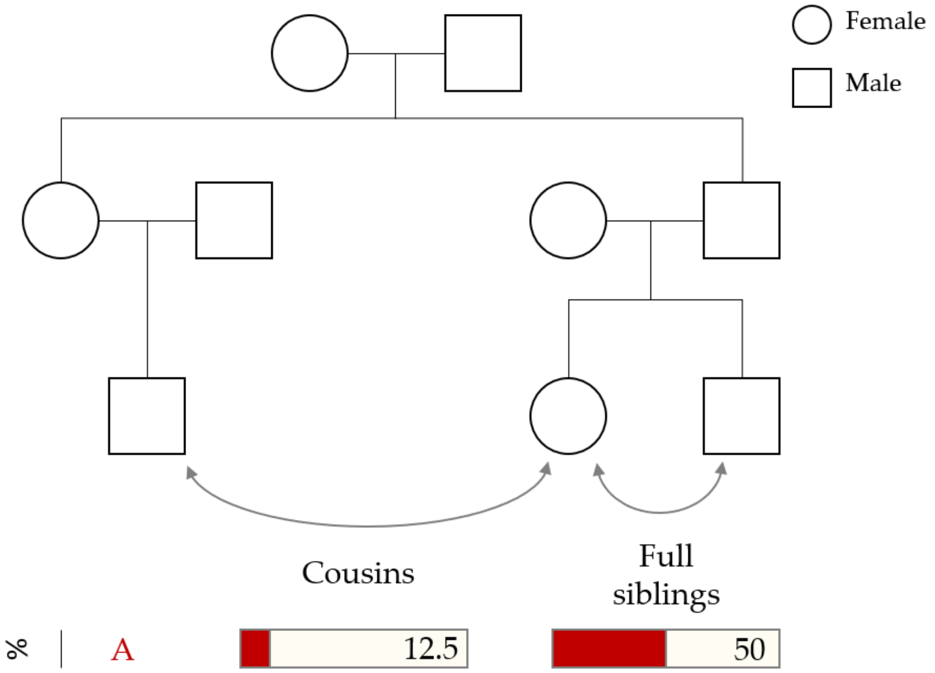

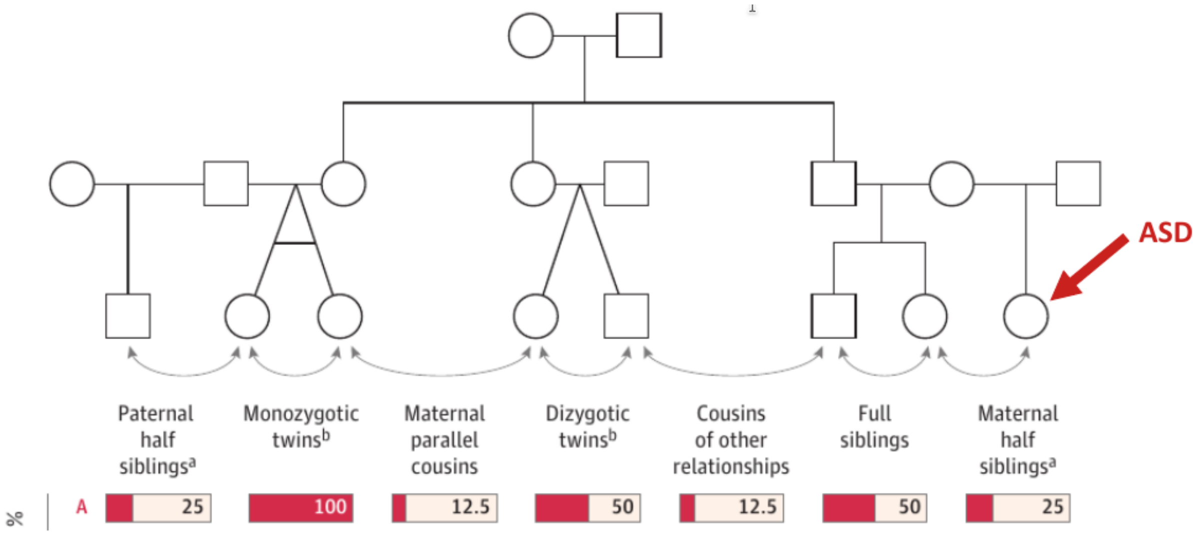

Note: Only full siblings and full cousins were included in the calculations.

© 2024 Sandin S et al. *JAMA Psychiatry*

eFigure 7. Profile likelihood for differences in heritability#

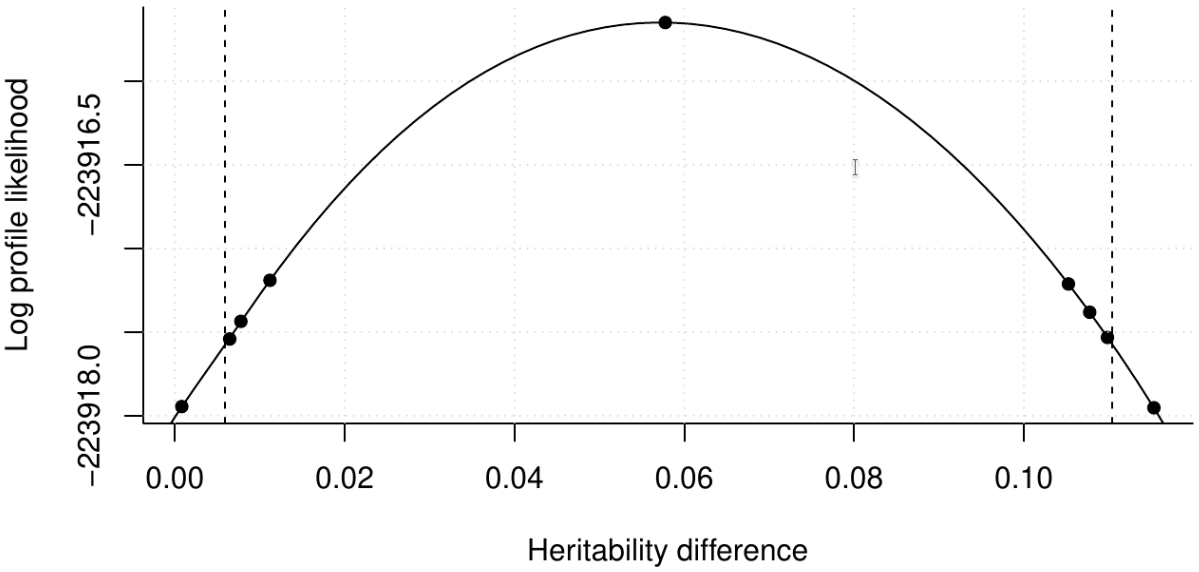

Note: The model incorporating birth-year (5 categories) and sex. The dots are points where the likelihood is evaluated and the line is an interpolation. The vertical dotted lines denotes statistical cut-offs corresponding to two-sided 95% confidence intervals.

© 2024 Sandin S et al. *JAMA Psychiatry*

eFigure 8 Plot of ASD prevalences for females who are exposed and not exposed (y-axis) plotted against the prevalence of the exposure (x-axis).

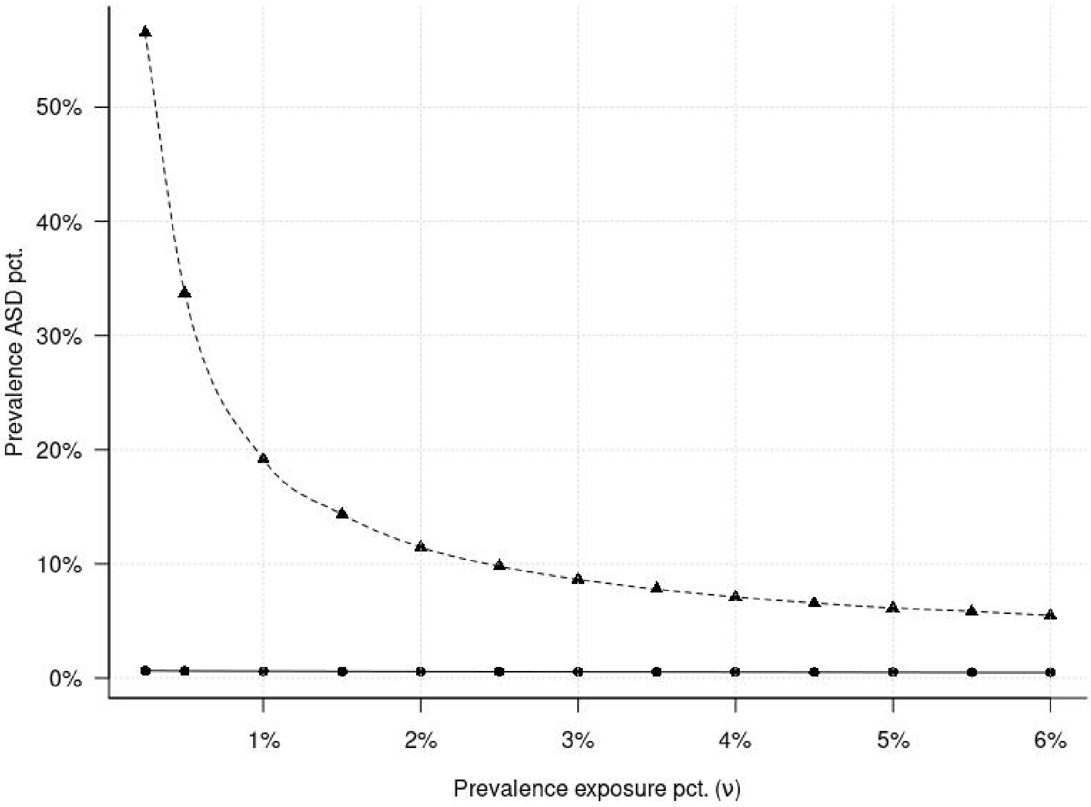

Another example, assuming 6% prevalence exposed would yield an increase by 0.98 SD. This correspond to a prevalence of $\phi(-2.58)=0.0050$, i.e. 0.5%, for the unexposed and $\Phi(-1.59)=0.0559$, i.e. 5.6%, for the exposed.

C.3. Summary of the scenarios in C1 and C2:

The examples from the previous sections show, within the model framework, consequences if a single female specific ASD causing factor drives the difference in heritability, or, if a set of different factors each perhaps of less impact explains the difference in heritability.

1. The first proposal assumes that there are many different underlying causes together generating a random effect (i.e., female specific residual). For this proposal to alone explain the difference in heritability between males and females, the set of causes building up the female variance would have to be responsible for at least 37% of the female ASD cases in the population.
2. For the second proposal to be true, that there is a single binary factor (e.g., *de novo* mutation or an environmental exposure), which explains the difference in heritability the factor must be very prevalent or extremely strong. Even with a prevalence as high as 6% the factor would have to be sufficiently strong to change the ASD prevalence in females in the general population from the observed 0.5% to 5.5%.

The two proposals may be considered extreme and a combination of causes could instead be true. Moreover, the total variance is not identifiable in absolute terms and only identifiable in relative terms between males and females under strong parametric assumptions. Hence, the above are all hypothetical causes and also assumes that the model assumptions are true.

© 2024 Sandin S et al. *JAMA Psychiatry*
