# Supplementary material for: Examining Sex Differences in Autism Heritability
Source: JAMA Psychiatry. 2024 Apr 17;81(7):673–80. doi: 10.1001/jamapsychiatry.2024.0525 (PMC11024771; doi:10.1001/jamapsychiatry.2024.0525)
Supplement: Supplement 2. — Data Sharing Statement [file jamapsychiatry-e240525-s002.pdf]

Data Sharing Statement

Sandin. Examining Sex Differences in Autism Heritability. *JAMA Psychiatry*. Published April 17, 2024. doi:10.1001/jamapsychiatry.2024.0525

Data
Data available: No
